# 4009 Magneto-electric nanoparticles (MENs) cobalt ferrite-barrium titanate (CoFe2O4–BaTiO3) for non-invasive neuromodulation

**DOI:** 10.1017/cts.2020.78

**Published:** 2020-07-29

**Authors:** Tyler Nguyen, Zoe Vriesman, Peter Andrews, Sehban Masood, M Stewart, Sakhrat Khizroev, Xiaoming Jin

**Affiliations:** 1 Indiana University School of Medicine; 2 Indiana University; 3 IUPUI; 4 University of Notre Dame; 5 University of Miami

## Abstract

OBJECTIVES/GOALS: Our goal is to develop a non-invasive stimulation technique using magneto-electric nanoparticles (MENs) for inducing and enhancing neuronal activity with high spatial and temporal resolutions and minimal toxicity, which can potentially be used as a more effective approach to brain stimulation. METHODS/STUDY POPULATION: MENs compose of core-shell structures that are attracted to strong external magnetic field (~5000 Gauss) but produces electric currents with weaker magnetic field (~450 Gauss). MENs were IV treated into mice and drawn to the brain cortex with a strong magnetic field. We then stimulate MENs with a weaker magnetic field via electro magnet. With two photon calcium imaging, we investigated both the temporal and spatial effects of MENs on neuronal activity both *in vivo* and in vitro. We performed mesoscopic whole brain calcium imaging on awake animal to assess the MENs effects. Furthermore, we investigated the temporal profile of MENs in the vasculatures post-treatment and its toxicities to CNS. RESULTS/ANTICIPATED RESULTS: MENs were successfully localized to target cortical regions within 30 minutes of magnetic application. After wirelessly applying ~450 G magnetic field between 10-20 Hz, we observed a dramatic increase of calcium signals (i.e. neuronal excitability) both *in vitro* cultured neurons and *in vivo* treated animals. Whole brain imaging of awake mice showed a focal increase in calcium signals at the area where MENs localized and the signals spread to regions further away. We also found MENs stimulatory effects lasted up to 24 hours post treatment. MEN stimulation increases c-Fos expression but resulted in no inflammatory changes, up to one week, by assessing microglial or astrocytes activations. DISCUSSION/SIGNIFICANCE OF IMPACT: Our study shows, through controlling the applied magnetic field, MENs can be focally delivered to specific cortical regions with high efficacy and wirelessly activated neurons with high spatial and temporal resolution. This method shows promising potential to be a new non-invasive brain modulation approach disease studies and treatments.

